# Proton Therapy, Magnetic Nanoparticles and Hyperthermia as Combined Treatment for Pancreatic BxPC3 Tumor Cells

**DOI:** 10.3390/nano13050791

**Published:** 2023-02-21

**Authors:** Francesca Brero, Paola Calzolari, Martin Albino, Antonio Antoccia, Paolo Arosio, Francesco Berardinelli, Daniela Bettega, Mario Ciocca, Angelica Facoetti, Salvatore Gallo, Flavia Groppi, Claudia Innocenti, Anna Laurenzana, Cristina Lenardi, Silvia Locarno, Simone Manenti, Renato Marchesini, Manuel Mariani, Francesco Orsini, Emanuele Pignoli, Claudio Sangregorio, Francesca Scavone, Ivan Veronese, Alessandro Lascialfari

**Affiliations:** 1 Istituto Nazionale di Fisica Nucleare, Sezione di Pavia, 27100 Pavia, Italy; 2 Dipartimento di Fisica “Aldo Pontremoli” and INFN (Sezione di Milano), Università degli Studi di Milano, 20133 Milano, Italy; 3 ICCOM-CNR, 50019 Sesto Fiorentino, Italy; 4 Dipartimento di Chimica, Università di Firenze and INSTM, 50019 Sesto Fiorentino, Italy; 5 Dipartimento di Scienze and INFN, Università Roma Tre, 00146 Roma, Italy; 6 Fondazione CNAO, 27100 Pavia, Italy; 7 Laboratorio Acceleratori e Superconduttività Applicata (L.A.S.A.), 20090 Segrate, Italy; 8 Dipartimento di Scienze Biomediche Sperimentali e Cliniche “Mario Serio”, 50134 Firenze, Italy; 9 Dipartimento di Fisica, Università degli Studi di Pavia, 27100 Pavia, Italy; 10 Fondazione IRCSS Istituto Nazionale dei Tumori, 20133 Milano, Italy; 11 INFN, Sezione di Firenze, 50019 Sesto Fiorentino, Italy

**Keywords:** magnetic nanoparticles, magnetic fluid hyperthermia, proton therapy, clonogenic survival, double strand breaks, pancreatic cancer

## Abstract

We present an investigation of the effects on BxPC3 pancreatic cancer cells of proton therapy combined with hyperthermia, assisted by magnetic fluid hyperthermia performed with the use of magnetic nanoparticles. The cells’ response to the combined treatment has been evaluated by means of the clonogenic survival assay and the estimation of DNA Double Strand Breaks (DSBs). The Reactive Oxygen Species (ROS) production, the tumor cell invasion and the cell cycle variations have also been studied. The experimental results have shown that the combination of proton therapy, MNPs administration and hyperthermia gives a clonogenic survival that is much smaller than the single irradiation treatment at all doses, thus suggesting a new effective combined therapy for the pancreatic tumor. Importantly, the effect of the therapies used here is synergistic. Moreover, after proton irradiation, the hyperthermia treatment was able to increase the number of DSBs, even though just at 6 h after the treatment. Noticeably, the magnetic nanoparticles’ presence induces radiosensitization effects, and hyperthermia increases the production of ROS, which contributes to cytotoxic cellular effects and to a wide variety of lesions including DNA damage. The present study indicates a new way for clinical translation of combined therapies, also in the vision of an increasing number of hospitals that will use the proton therapy technique in the near future for different kinds of radio-resistant cancers.

## 1. Introduction

To fight cancer, medicine has multiple tools at its disposal including, e.g., surgery, X-ray radiation therapy, chemotherapy, hadron therapy, brachytherapy, and immunotherapy. Particularly, in recent years, research has introduced new strategies against cancer, especially in the field of precision medicine [1]. Scientists devoted strong effort towards the use of nanotechnology and nanomaterials, which, after reaching cancer cells, for instance, can release chemotherapeutic drugs [2] and/or cause an increase in temperature if subjected to an external stimulus. This last case can be realized in different ways, for example with the use of magnetic nanoparticles (MNPs) that release heat locally under the action of an alternating magnetic field (AMF), thus resulting in anti-tumor effects [3,4]. In particular, the increment of tumor cells’ temperature up to 42–45 °C, also referred to as Magnetic Fluid Hyperthermia (MFH), reduces or stops the cancer growth, thanks to the induction of cell apoptosis. This type of hyperthermia has fewer side effects compared to whole body [5] and regional [6] methods, as healthy tissues are being spared. The MFH technique has been introduced clinically in Europe to treat the glioblastoma multiforme brain tumor, while, at the moment, FDA-approved clinical trials are performed on prostate carcinoma [7], especially in the USA. The (thermal) energy losses are induced by hysteresis/relaxation effects of magnetic nanoparticles when exposed to an AMF [8]. The nanoparticle size distribution and concentration are crucial parameters to guarantee a homogeneous and sufficiently high temperature increment, taking into account that temperatures of the order of 42–45 °C have to be reached quite rapidly (within a few minutes) to have an efficient therapeutic treatment. Too small nanoparticles (mean diameter d < 10 nm) usually do not have a high enough Specific Adsorption Rate (SAR) to reach the therapeutic temperature window and are eliminated through the kidneys, whereas too big nanoparticles (d > 40–50 nm) are phagocytized and transported to the liver by macrophages, since they are recognized as exogenous objects [9]. Moreover, to guarantee the patient safety, the hyperthermic treatment requires the use of a combination of AMF amplitude and frequency values which does not cause side effects [10], and the injection of a minimum dose of MNPs. Results of clinical safety investigation led to the Brezovich criterion [10] and consequently to the application of AMFs of amplitude 10–20 kA/m and frequency 100–500 kHz [11,12]. On the other hand, it is worth mentioning that other possible biological effects, e.g., immune response [13], could occur, but this has to be investigated specifically (it is not the aim of the present investigation).

For more than a decade, to provide an increased effectiveness of the treatment, combined therapies have often been used, with the aim of inducing an additive or synergistic effect able to obtain a higher killing effect of the tumor cells. Moreover, to have tumor growth control, the dose required in radiotherapy and the drug loading in chemotherapy can be decreased, for example, by associating a combined hyperthermia treatment. Previous studies, in fact, reported that the combination of these treatments can improve the control of tumor growth at the local level and enhance the overall survival rate in some tumors [14]. However, this approach is often not efficient enough for tumors that are inherently resistant to conventional chemotherapy and radiotherapy, e.g., pancreatic carcinoma, which is one of the aggressive cancers with poor prognosis and a low survival rate (of about 5 years [15,16]). In this case, highly and inherently conformal radiation therapy techniques such as hadron therapy (HT) (that makes use of charged particles such as protons, carbon ions and helium ions, and, for this reason, is also called Particle Therapy) turn out to be promising alternative options to conventional radiotherapy. Indeed, HT offers several advantages over conventional photon radiotherapy, including a more accurate dose distribution and, in the case of carbon ions, a higher amount of damage induced on the tumor [17]. By the end of 2021, about 325,000 patients had been treated worldwide with Particle Therapy: close to 280,000 with protons, about 42,000 with C-ions and about 3500 with He and other ions (Particle Therapy Co-Operative Group [18]). It is worth noticing that, unfortunately, for a long time, one of the most striking disadvantages of particle (also proton) therapy has been the high cost of its technical realization and operation: large and expensive cyclotrons or synchrotrons were needed to accelerate protons and heavier ions until they reached the energy levels required for the treatment of deep tumors. Instead, nowadays, thanks to advances in technology, relatively small hospital proton accelerators have been designed and installed to overcome the above reported limits, thus allowing to offer the benefits of proton therapy (PT) to an increasing number of patients.

Within this framework, in our previous work [19], carbon ion irradiation was delivered to BxPC3 pancreatic tumor cell cultures, combined with MNPs administration and hyperthermia treatment. Important results included: (a) a killing (toxic) additive effect of about 50% on clonogenic survival (CS) due to the cellular uptake of MNPs being observed at all carbon ions doses; and (b) a further additive effect of about 15–30% on CS due to hyperthermia also being obtained.

Taking into account these very promising results, we present here an investigation aimed at determining the effect of proton irradiation combined with MNPs uptake effect and hyperthermia and at deepening the role of other biological effects. Particularly, for proton and photon irradiation, we determined the cells’ response to the treatment by means of the clonogenic survival assay, the induction of DNA Double Strand Breaks, the ROS formation, the RBE, cell cycle and cell invasion. Moreover, as a completion of our first study [19], for all irradiation modalities (with protons, photons and, old data, carbon ions), we evaluated the effects of the single irradiation and/or the combined-hyperthermia treatment on cell cycle variations, tumor cells invasion and cellular damage, and we measured the dose enhancement factors of the different radiation beams. As the main results, a synergistic effect of proton therapy, MNPs uptake and hyperthermia treatment on clonogenic survival was observed, until the level of a 65–80% increase of killing ability with respect to the single proton therapy. This result has to be compared with the previous one [19] on carbon ion irradiation combined with hyperthermia where a similar effect, but additive, was singled out.

## 2. Materials and Methods

### 2.1. Magnetic Nanoparticles

MNPs were prepared by the same synthesis procedure described in [19]: they consist of monodisperse spherical MNPs with a mean diameter of 19.2 ± 3.6 nm, covered with Meso-2,3-dimercaptosuccinic acid (DMSA). The characterization of the samples was performed by using standard techniques (TEM, XRD, DLS magnetometry, calorimetric measurements). The experimental results confirmed that the morphological, structural, magnetic and hyperthermic properties of these samples are in the range of values previously reported for MNPs used in past experiments [19].

### 2.2. Cell Culture

BxPC3 cells of human pancreatic adenocarcinoma, supplied by the ICLC (Interlab Cell Line Collection, Genova, Italy), are cultivated at 37 °C and 5% CO${}_{2}$ using RPMI 1640 media (Roswell Park Memorial Institute, Sigma-Aldrich, St. Louis, MO, USA) supplemented with 10% fetal bovine serum and 50 μg/mL of gentamicin (Sigma-Aldrich). The value of plating efficiency (PE) is about 50%.

### 2.3. Clonogenic Toxicity

BxPC3 cells in exponential growth were incubated with MNPs (50 μg/mL) for 48 h. Afterwards, aliquots of treated MNPs cells were exposed to hyperthermia treatment (42 °C for 30 min); then, the cells were harvested by trypsinization (using 0.25% trypsin-EDTA), counted and plated into T25 flasks at appropriate numbers for colony-forming assays, and incubated at 37 °C. After 14 days of incubation, the samples were fixed with 70% ethanol and stained with 10% Giemsa solution; colonies were counted, and the plating efficiency was determined.

### 2.4. MNPs’ Cellular Uptake

Forty-eight hours after incubation with a concentration of 50 μg/mL, elemental iron (Fe) was quantitatively measured by inductively coupled plasma optical emission spectrometry (ICP-OES) with iCAP 6200 Duo upgrade (Thermofisher, Waltham, MA, USA). After sampling, digestion with nitric acid at room temperature (T = 22 °C) was performed only on a fraction of the available volume, since the solution appeared to be homogeneous after the vortexing. The concentration of Fe contained in the cells was then converted to the number of MNPs uptaken per cell by considering both the size of the nanoparticles obtained from TEM imaging and the total number of cells involved. From the experimental results, the mean uptake for the proton experiment (computed by averaging the results of the ICP measurements performed for six different samples) is ∼8.5 pg(Fe)/cell.

### 2.5. Irradiation

Cell irradiation with protons was performed using the synchrotron-based clinical scanning beams (fixed horizontal beam line) at Centro Nazionale di Adroterapia Oncologica (CNAO, Pavia, Italy). The flasks were placed vertically inside a water phantom put in the isocenter on the treatment table, at the depth of 15 cm, corresponding to the mid spread-out Bragg peak (SOBP). The SOBP (6 cm width, from 12 to 18 cm depth in water) was achieved with active beam energy modulation, using 16 different energies (131.5–164.8 MeV). The proton dose-averaged linear energy transfer (LET) in the mid SOBP, evaluated with a Monte Carlo FLUKA simulation, was 3.6 kev/μm. Cell samples were irradiated in the dose range 0–4 Gy.

Photon beam irradiation was performed using a 6 MV linear accelerator (Varian Clinac 2100C, Varian Medical System, Palo Alto, CA, USA) at the Istituto Nazionale dei Tumori di Milano, with different doses (0–7 Gy). The flasks containing the cells were irradiated using a vertical beam 20 × 20 cm${}^{2}$ field, placing them horizontally at the isocenter in a water phantom at 5 cm depth.

### 2.6. Clonogenic Assay

BxPC3 cells were plated in T25 flasks (about 6 × 10${}^{5}$ cell/flask) and, after 48 h, some cell samples were incubated with MNPs (50 μg/mL) for 48 h at 37 °C. Afterwards, the cells were exposed to radiation at different doses and, in about 1 h, to hyperthermia treatment. After irradiation, the cells were harvested by trypsinization, counted and re-plated into five T25 flasks for each dose at appropriate numbers for colony-forming assays and incubated for 14 days. For the hyperthermia treatments, the irradiated cells with MNPs were trypsinized, centrifuged (1500 rpm, 10 min) and the cellular pellets (about 2 × 10${}^{6}$ in 0.1 mL medium) were transferred into 0.2 mL polypropylene Eppendorf mini-tubes. The heating was applied for 30 min at 42 °C (see [19]). Following this hyperthermia treatment, the cellular samples underwent the same protocol for the determination of cell survival at 14 days. All the samples were fixed with ethanol and stained with 10% Giemsa solution, and colonies consisting of more than 50 cells were scored as survivors. The surviving fraction (SF) was calculated as the plating efficiency (PE) of the irradiated samples divided by the PE of the unirradiated sample.

### 2.7. Double Strand Breaks (DSBs) Studies

The single/combined effect of proton irradiation and magnetic fluid hyperthermia in the induction of DNA damage (Double Strand Breaks, DSBs) was measured. The BxPC3 cells were seeded in slide flasks (Thermo Fisher Scientific, Waltham, MA, USA) and collected 6 h and 24 h later. The kinetics of DSBs repair was evaluated by means of γ-H2AX and 53BP1 foci formation determined through immunofluorescence analysis (Zeiss Axio Imager M1 microscope). The frequencies of both DNA damage marker foci per cell were scored on 100 nuclei in 4 (photons) or 5 (protons) independent experiments.

### 2.8. Hyperthermia (Hyp) Measurements

Hyperthermia (Hyp) was realized by means of a thermalization system surrounding the samples (responsible for 60% of the temperature increase ΔT) assisted by Magnetic Fluid hyperthermia (the remaining 40% ΔT) [19]. Magnetic hyperthermia experiments were performed using MagneTherm by Nanotherics, working with an AMF of frequency 109.8 kHz and an amplitude of 15.6 kA/m. The temperature was increased from ∼37 °C to ∼42 °C (ΔT = 5 °C). The temperature of the samples was measured using an Optocon optical fiber thermometer positioned at the center of the sample placed inside an Eppendorf PCR Tube (for more information, see [19]).

### 2.9. Detection of Reactive Oxygen Species (ROS), Cell Cycle Analysis and Cell Invasion

#### 2.9.1. Detection of Reactive Oxygen Species (ROS)

2${}^{\prime }$,7${}^{\prime }$-Dichlorofluorescein diacetate was used in the quantitative assay to measure oxidative stress (Reactive Oxygen species production) in nanoparticle-treated cells/radiation exposed cells.

#### 2.9.2. Cell Cycle Analysis

For cytofluorimetric analysis, the cells were treated with Ribonuclease A for 30 min at 37 °C and then stained with propidium iodide for about 12–14 h. The proportion of cells at different phases was gated and calculated using the ModFit Lt software.

#### 2.9.3. Cell Invasion

Cell invasion is measured using the QCM EC Matrix Cell Invasion Assay (Merck Millipore) with an 8 µm pore size polycarbonate membrane, considering the ratio between invading cells and the total number of cells initially seeded, and then normalized to the control ones.

For more experimental details on the detection of reactive oxygen species, cell cycle and cell invasion, see Supporting Information.

## 3. Results and Discussion

A summary of the experiments conducted on the BXPC3 cell line is provided below (see Table 1).

**Figure 1:**
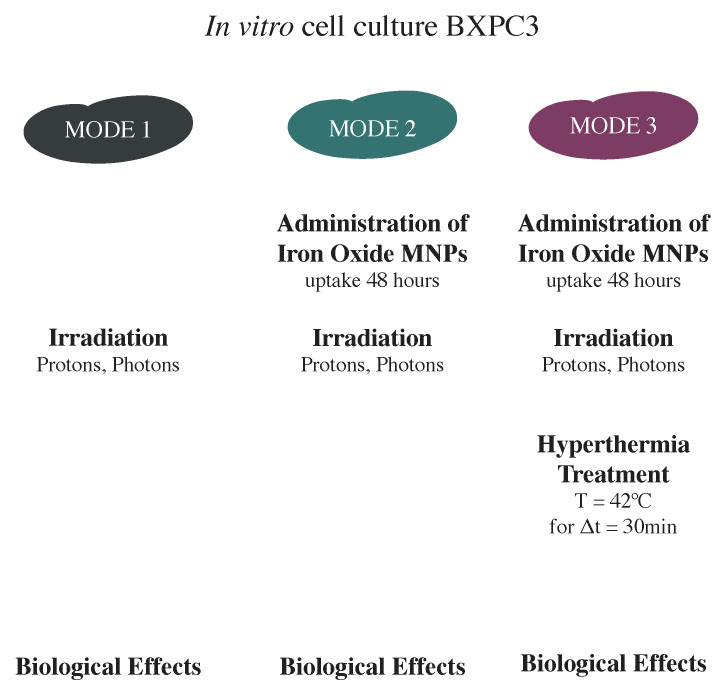
Description of the three different treatment modes used during the experiments: simple irradiation (*Mode 1*), iron oxide magnetic nanoparticles (MNPs) administration and irradiation (*Mode 2*) and MNPs administration plus irradiation and subsequent hyperthermia (*Mode 3*).

In Figure 1, we sketch the general experimental treatment protocol, which consists of three different treatment *modes*: (1) irradiation alone (protons, photons); (2) MNPs administration and irradiation; (3) MNPs administration plus irradiation and subsequent hyperthermia. The biological effects of the different treatment modes were assessed: (i) by using a clonogenic assay after two weeks; (ii) by estimating the number of non-repairable double strand breaks (DSBs) per cell after 6 h and 24 h; (iii) by evaluating other (radio)biological effects, i.e., ROS production, cell cycle variations and cell invasion ability modification.

### 3.1. MNPs and Hyperthermia Cell Toxicity

The plating efficiency of BxPC3 cells decreases from about 50% to 23% when MNPs are added, due to the MNPs toxicity at 14 days. A further decrease of PE to about 6% is observed when hyperthermia is also applied. Therefore, hyperthermia in cells treated with MNPs shows a further effect of cell mortality compared to treatment with MNPs alone. Similar results were reported in literature for other cells line (see SI). There are few data in the literature on cell MNP toxicity in BxPC3 cells and almost no data on cytotoxicity measured by clonogenic assay (except [19]). In the work of Hannon et al. [20], the cell toxicity was evaluated using three Stain Tests (the multiparametric analysis, i.e., cell count, nuclear membrane permeability, lysosomal permeability) after 72 h treatment of nanoparticles, and the results showed no significant changes compared to the untreated control. This is in agreement with our previously reported data [19]. In fact, the cytotoxicity measured by a Trypan Blue assay was not significantly affected by the presence of MNPs after 48–72 h of treatment, thus allowing for concluding that, for short amounts of time (<72 h), no cytotoxicity was detected, in agreement with most literature results.

### 3.2. Clonogenic Survival: Proton and Photon Irradiation Experiments

**Figure 2:**
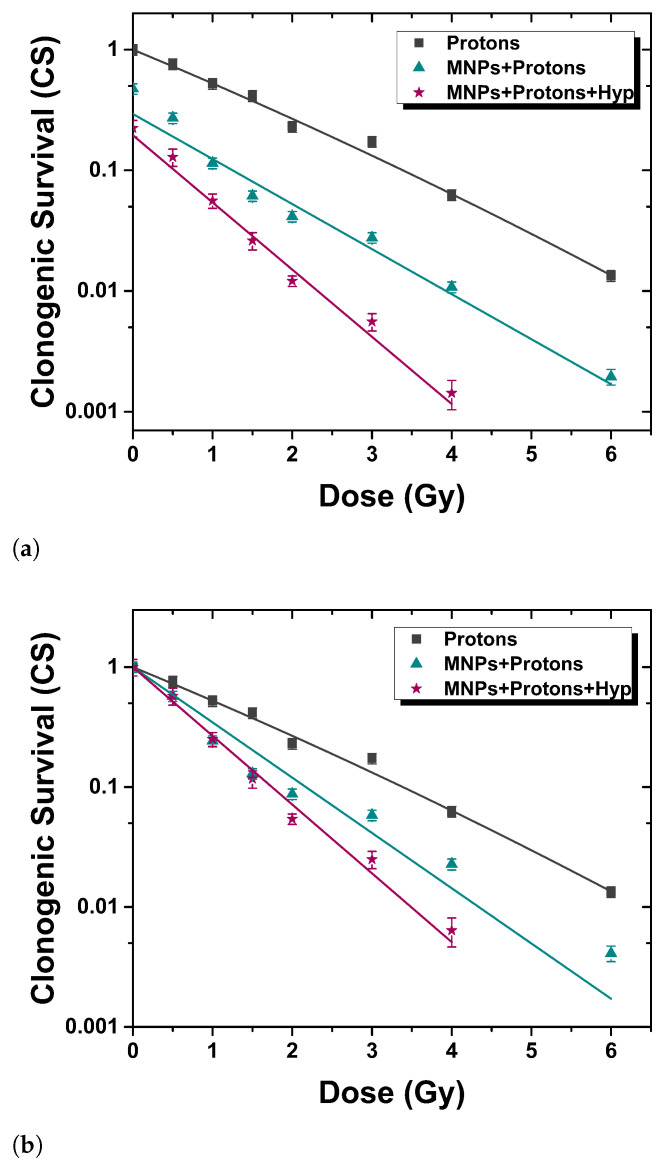
Clonogenic survival of BxPC3 cells culture for three different protocols (see text): Proton therapy (PT) only (gray squares, *Mode 1*), PT+MNPs administration (teal triangles, *Mode 2*) and PT+MNPs administration+Hyp (plum stars, *Mode 3*). A synergistic effect of MNPs and of MNPs+Hyp is noted. (**a**) not normalized curves; (**b**) normalized curves.

**Figure 3:**
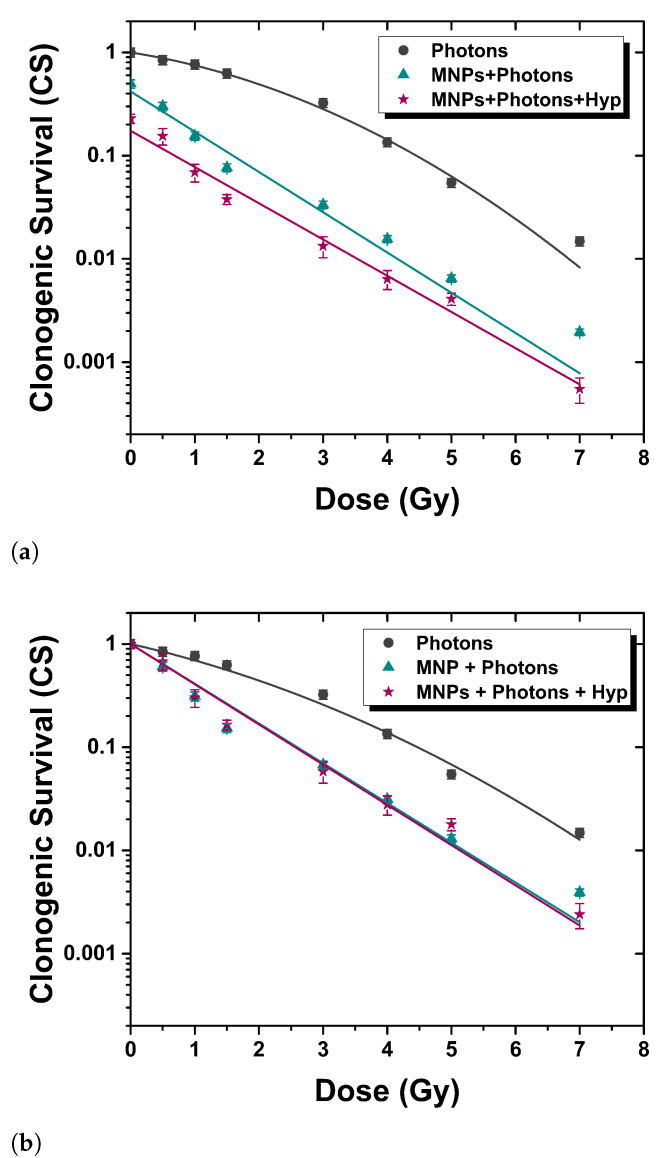
Clonogenic survival of BxPC3 cells culture for three different protocols (see text): photon therapy only (gray squares, *Mode 1*), photon therapy+MNPs administration (teal triangles, *Mode 2*) and photon therapy+MNPs administration+Hyp (plum stars, *Mode 3*). (**a**) not normalized curves; (**b**) normalized curves.

The assessment of the effect of the combined treatment was performed using a clonogenic assay, i.e., cytotoxicity for long amounts of time. Figure 2 and Figure 3 show the clonogenic surviving fraction of BxPC3 cells treated with three different protocols (*Modes*, see Figure 1): (i) irradiation alone (gray squares), (ii) proton/photon irradiation after the administration of magnetic nanoparticles for 48 h (teal triangles) and (iii) proton/photon irradiation after the administration of MNPs combined with 30 min of hyperthermia treatment at 42 °C (plum stars). The results for each protocol were averaged over five independent experiments for protons, and four independent experiments for photons. The surviving curve of radiation alone (*Mode 1*) was fitted according to the linear quadratic model, CS = exp(−αD −βD${}^{2}$), where CS is the clonogenic survival and *D* the delivered dose. The use of this fitting model was necessary due to the typical “shoulder” displayed by the experimental data at low (<3 Gy) doses. The α and β values obtained are: (i) for protons, $\alpha_{Mode\;1}$ = (0.63 ± 0.05) Gy${}^{-1}$, $\beta_{Mode\;1}$ = (0.015 ± 0.010) Gy${}^{-2}$, (ii) for photons, $\alpha_{Mode\;1}$ = (0.22 ± 0.06) Gy${}^{-1}$, $\beta_{Mode\;1}$ = (0.07 ± 0.01) Gy${}^{-2}$. Data obtained after *Modes 2* and *3* treatments were fitted to the function CS = CS${}_{0}\cdot$exp(−αD), where the parameter CS${}_{0}$ represents the clonogenic survival of cells treated with MNPs and not irradiated. The α values obtained are: (i) for protons, $\alpha_{Mode\;2}$ = (1.06 ± 0.07) Gy${}^{-1}$, $\alpha_{Mode\;3}$ = (1.32 ± 0.04) Gy${}^{-1}$; (ii) for photons, $\alpha_{Mode\;2}$ = (0.90 ± 0.09) Gy${}^{-1}$, $\alpha_{Mode\;3}$ = (0.81 ± 0.06) Gy${}^{-1}$. It is worth noting that the data obtained in *Modes 2* and *3* show a different radiation dose-dependence: the clonogenic survival decreases exponentially with the dose, without any “shoulder”.

#### 3.2.1. Proton Irradiation

Referring to Figure 2, it is evident that the addition of MNPs leads to a significant decrease of the *CS* and that both MNPs uptake alone and MNPs uptake plus Hyp grant a killing effect on tumor cells, which is synergistic with irradiation. This is evident (particularly for the curves normalized to zero doses values, Figure 2b) from the different laws followed by the experimental data in the case of simple irradiation (linear quadratic fit, *Mode 1*) and irradiation coupled to MNPs uptake (linear model fit, *Mode 2*) or to MNPs uptake+Hyp (linear model fit, *Mode 3*). Moreover, one can observe the following: (i) the synergy of irradiation with MNPs administration suggests an effect of radiosensitization of the MNPs; (ii) the linear model curve changes by passing from *Mode 2* to *Mode 3* results. This effect suggests that Hyp gives a further synergistic effect with respect to irradiation and MNPs administration. Literature results confirm the radiosensitization induced by nanoparticles. Polf et al. [21] present a study of changes in the efficacy of proton radiotherapy for human prostate carcinoma cells containing gold nanoparticles, thus demonstrating a decrease in cell survival when AuNPs are uptaken by cells. In the study of Rashid et al. [22], the radiosensitization effects induced by different types of nanoparticles on human colon carcinoma cells irradiated with 150 MeV proton beam were demonstrated by the reduction of cell survival.

#### 3.2.2. Photon Irradiation

Figure 3 summarizes the results for the conventional photon radiotherapy combined or not with magnetic nanoparticles administration and hyperthermic treatment. Similar results have also been reported in [19] compared to carbon ion irradiation. As in the case of protons, the MNPs administration alone (*Mode 2*) and combined with Hyp (*Mode 3*) gives a synergistic effect with irradiation, as from the change of the law satisfied by the curve CS vs. D. Important differences with PT are: (a) the slope of the CS vs. D curve is the same for *Mode 2* and *Mode 3* (see Figure 3b), i.e., Hyp is additive with respect to MNPs administration; (b) the absolute values of α and β are not the same, as expected for the case of different radiations. The results of literature agree with ours. Ahmad et al. [23] report that the addition of several types of nanoparticles create a significant decrease in cell survival in two cell lines (U87 and MCF-7) after X-rays irradiation. An inherent increased DNA damage and decreased survival were observed with 23.5 μg/mL SPIONs in the MiaPaCa2 pancreatic cell line, for a 24 h incubation time through a preclinical 225 kVp exposure [24].

### 3.3. Double Strand Breaks Studies

As a measure of the single/combined effect of hyperthermia and photons/protons in the induction of DNA damage, the kinetics of DNA double strand breaks (DSBs) rejoining has been evaluated by means of γ-H2AX and 53BP1 foci formation by immunofluorescence analysis (see Figure S4 in SI for proton irradiation). Both γ-H2AX (phosphorylation at Ser-139) and 53BP1 are well validated markers of DNA double-strand breaks [25,26].

**Figure 4:**
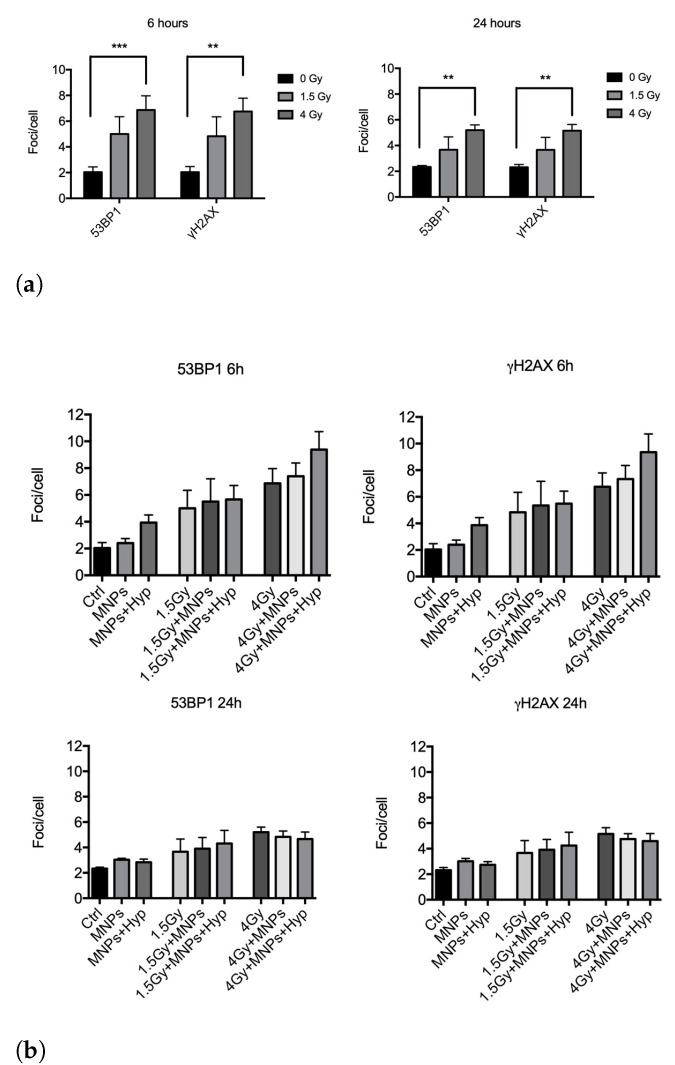
Analysis of 53BP1 and γ-H2AX foci induction in BxPC3 pancreatic tumor cells after 6 h and 24 h from the exposure to: (**a**) 1.5 and 4 Gy of photon irradiation alone (*Mode 1*); (**b**) no dose (control, Ctrl) or photon irradiation in combination with MNP uptake (*Mode 2*) and MNPs uptake + Hyp (*Mode 3*). ** indicates *p*-value *p* < 0.01, *** indicates *p* < 0.005 (one-way ANOVA and Tukey’s multiple comparison post-test).

Regarding the 6 MV photon beam irradiation, we collected and analyzed results from four independent experiments after exposure of BxPC3 pancreatic tumor cells to 1.5 and 4 Gy and harvested at 6 h and 24 h in the three different treatment modes (see Figure 1). In Figure 4, we report “foci/cell” values at different doses. We found that photons alone (*Mode 1*) significantly increased the number of DSBs with respect to control, i.e., untreated, samples (Figure 4a), an effect still visible at 24 h from exposure for both antibodies. An increase of DSBs number by adding to irradiation also the MNPs uptake (*Mode 2*) and MNPs uptake+Hyp (*Mode 3*) was observed at 6 h (Figure 4b, upper part), while, at 24 h, no effect was visible anymore.

**Figure 5:**
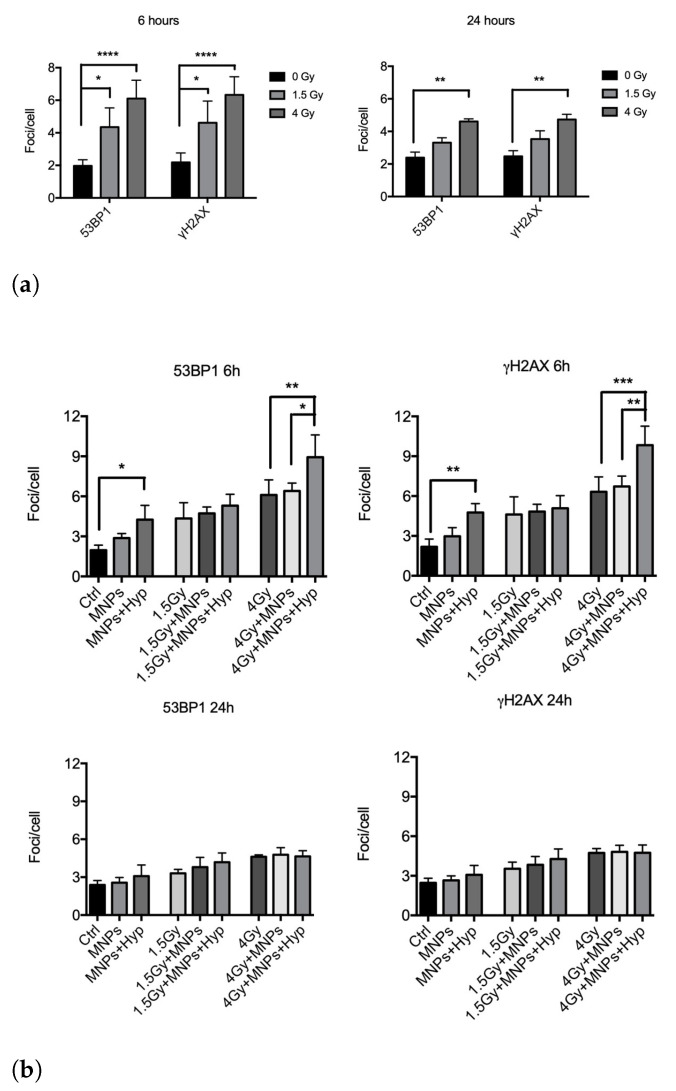
Analysis of 53BP1 and γ-H2AX foci induction after 6 h and 24 h, in BxPC3 pancreatic tumor cells, from: (**a**) the exposure to 1.5 and 4 Gy of proton irradiation alone; (**b**) the same proton irradiation in combination with MNP uptake and/or Hyp. * indicates *p*-value *p* < 0.05, ** indicates *p* < 0.01, *** indicates *p* < 0.005, **** indicates *p* < 0.0001 (one-way ANOVA and Tukey’s multiple comparison post-test).

Similar experiments were carried out with proton irradiation (five independent experiments), again exposing cells to 1.5 and 4 Gy doses. Here, proton irradiation alone (*Mode 1*) induced a significant increase of DSBs at both doses for the 6 h harvesting time; such increase was still detectable at 24 h mainly for the highest dose. For 0 and 4 Gy doses, the Hyp treatment added to irradiation and MNPs uptake (i.e., *Mode 3*) increased the number of DSBs at 6 h compared to samples undergoing *Mode 1* and *Mode 2* treatments (Figure 5b, upper part). At 1.5 Gy, this increase was not observed. At 24 h (Figure 5b, lower part), effects of *Mode 3* treatment were no longer visible.

Overall, these experiments seem to indicate, in agreement with the clonogenic assay, differences in the quality of DNA damage between the two types of radiations, when coupled to MNPs administration and Hyp. Interestingly, in our previous experiments with carbon ion beams [19], we detected an increase in DSBs induction after treatment for both 0.75 and 1.5 Gy only at 6 h, suggestive of a repair which occurs for longer times [27]. This seems to be in contrast with the expected higher complexity of DNA damage in the case of carbon ions (and therefore longer persistence and less reparability of DNA lesions), responsible for the RBE as calculated with the clonogenic assay (RBE carbon ions: 3.5, RBE protons: 1.3). Since apoptosis is not different among the types of radiations used (Figure S11 in SI), differences at 24 h in the persistence of DSBs between the different types of radiation might not be solely ascribed to repair processes, but also, in the case of high irreparable damage, to some forms of cytotoxicity, other than apoptosis, which eliminate highly damaged cells. As for carbon ions [19], also for protons, the *Mode 3* treatment was able to increase the induction of DNA DSBs produced by radiations (an effect was not detected for photons plus MNPs and hyperthermia). This could be partly explained by the prevalent contribution of HR (Homologous Recombination) over NHEJ (Non-Homologous End Joining) in radiosensitization effects, probably affecting the processing of a subset of DNA DSBs lesions [28]. In this respect, at the molecular level, it was shown that Hyp (T > 41 °C) did inhibit HR in human and mouse cells [29].

### 3.4. Relative Biological Effectiveness and Dose Enhancement Factor

We quantified the relative biological effectiveness (RBE) of protons for BxPC3 tumor cells (and compared it with photon irradiation), and the dose enhancement factor (DEF), defined as the ratio between the radiation doses used alone and in conjunction with the MNPs (50 μg/mL) in order to obtain the same biological endpoint (i.e., the same survival level). We found that the proton RBE value at 10% of survival is 1.27±0.3. This value is higher than 1.1, which is conventionally used for therapeutic proton beams [30]; however, some reviews [31,32] pointed out significant variations for in vitro values of proton RBE. It is worth noting that, in our previous study, we showed that, at 10% of clonogenic survival, the RBE was equal to about 3.5 for BxPC3 cells irradiated with carbon ions (LET = 45 keV/µm) [19]. More details on RBE are found in SI.

As concerns the DEF (more details in SI), we analyzed data from photons and protons, together with data from our old study [19] on carbon ions. DEF values for photons, protons and carbon ions, respectively, are: (i) 2.8 ± 0.3, 2.5 ± 0.3 and 2.0 ± 0.2 at 10% of survival; (ii) 1.8 ± 0.2, 1.7 ± 0.2 and 1.6 ± 0.2 at 2% of survival. The experimental results indicate that the MNPs induce radiosensitization effects, and the DEF values at 10% of survival show that the presence of these nanoparticles caused damage to the cells with all types of radiations. Figure S5 in SI shows the comparison of DEF at two survival levels for all types of radiation beams.

### 3.5. Detection of Reactive Oxygen Species, Cell Cycle Analysis and Cell Invasion

**Figure 6:**
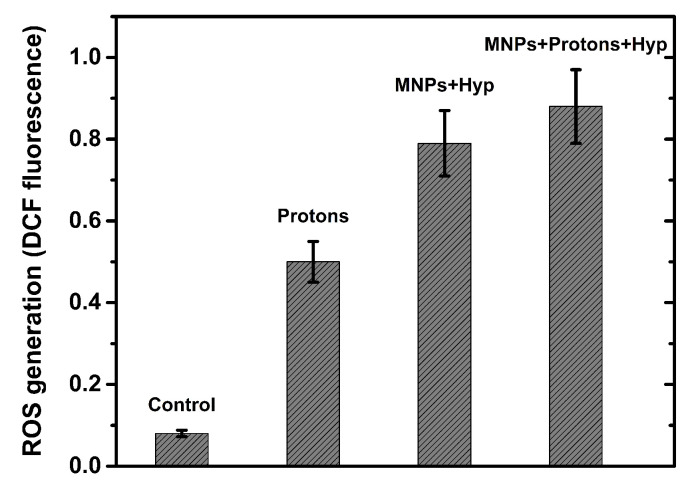
Flow cytometry analysis of BxPC3 cells to detect ROS induction after proton irradiation, MNPs and hyperthermia. Control: untreated cells; Protons: cells irradiated with 4 Gy of protons; MNPs+Hyp: cells with MNP-50 μg/mL and hyperthermia treatment; MNPs+Protons+Hyp: cells with MNP-50 μg/mL, irradiated with 4 Gy of protons and hyperthermia treatment. Mean ± SD (2 experiments).

The *reactive oxygen species* (ROS) generation data are reported in SI, Figures S6–S9, for different cell samples. As an example, we report in Figure 6 the ROS generation after proton irradiation, MNPs administration and hyperthermia treatment. The exposure of BxPC3 cells to radiation alone (either protons or photons or carbon ions) resulted in an increase in the level of ROS. A greater increase occurs when BxPC3 cells are incubated with MNPs (at concentration of 50 μg/mL); a further increase of ROS level is generated by successive Hyp treatment. Therefore, hyperthermia (also MFH) has been shown to elevate the production of ROS, which also contributes to cytotoxic cellular effects and to a wide variety of lesions including DNA damage. More details on ROS are found in SI.

**Figure 7:**
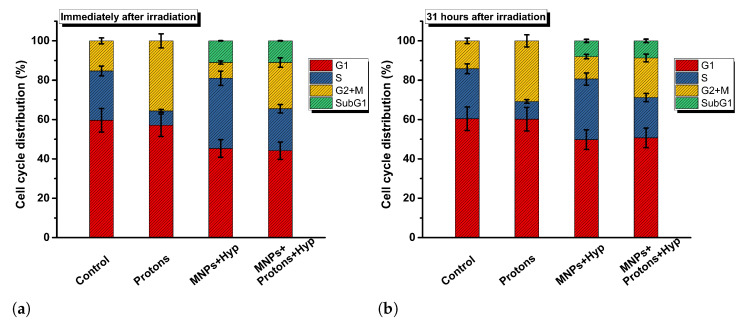
Cell cycle phase distribution of BxPC3 cells after proton irradiation, MNPs and hyperthermia. Control: untreated cells; Protons: irradiated cells; MNPs+Hyp: cells with MNP-50 μg/mL and hyperthermia treatment; MNPs+Protons+Hyp: cells with MNP-50 μg/mL, irradiation and hyperthermia treatment. Cell cycle phases are evaluated immediately after (**a**) and 31 h after (**b**) the irradiation.

As for *cell cycle*, the data reported in Table S1 of SI show that there are no variations in the values of the cell phases in samples treated with MNPs for 48 h (50 μg/mL, without irradiation and hyperthermia treatments) and untreated (control). This result indicates that MNPs incorporated into BxPC3 cells do not alter the cell cycle. Similar results have been found in some works with different cell lines (see Refs. [33,34,35]). Exposure of BxPC3 cells to a dose of 2 Gy of carbon ions (data from our old work [19]), 4 Gy of protons (see Figure 7) or 5 Gy of photons resulted in an increase of cells in the G2/M phase. Thus, our results show that irradiation with carbons, protons and photons induce “G2/M cell cycle arrest” [36], indicating a high level of DNA damage. Cells with unrepaired or poorly repaired DNA damage can persist in the G2/M phase, leading to genomic instability, cell death and therefore to an inhibition of cell proliferation. More details are reported in SI.

On the other hand, the results of the *cell cycle analysis* after MNPs and hyperthermia treatments, without irradiation, show an increase of the S phase, about 36% compared to a value of about 25% for the samples without MNP and hyperthermia treatments (see Figure S10 in SI). The cell samples treated with MNPs+hyperthermia and MNPs+radiation+hyperthermia (see Figure S11 in SI) show a percentage of apoptotic cells around the value of 8–12% (immediately after irradiation and 24–34 h post irradiation); this increase appears to be due to the combination of the treatments.

**Figure 8:**
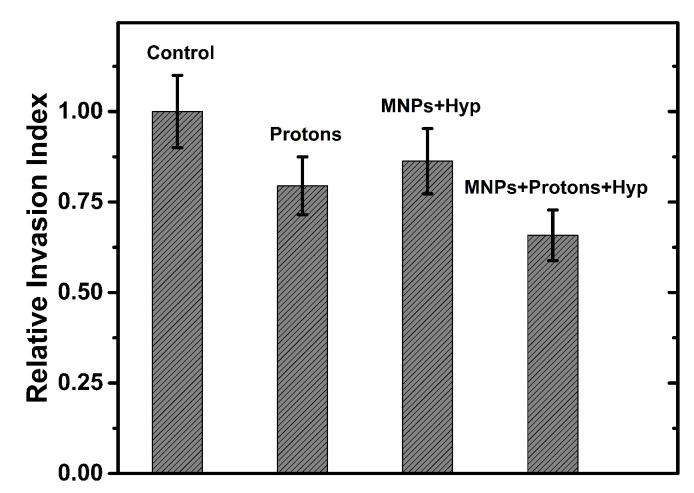
The relative invasion index of BxPC3 cells exposed to protons, and treated or not with MNPs and hyperthermia. Control: untreated cells; Protons: cells irradiated with 4 Gy of protons; MNPs+Hyp: cells with MNP-50 μg/mL and hyperthermia treatment; MNPs+Protons+Hyp: cells with MNP-50 μg/mL, irradiated with 4 Gy of protons and hyperthermia treatment. Error bars represent one standard error of the mean of two experiments.

*Cell invasion* is related to cell migration and defines the ability of cells to become motile and to navigate through the extracellular matrix within a tissue or to infiltrate neighboring tissues. Cancer cells that become invasive may disseminate to secondary sites and form metastases. Thus, tumor cell invasion is an essential step of cancer progression that is associated with an enhanced capability of tumor cells to degrade extracellular matrix components. In our case (BxPC3 cells), it is noted first that no effect on cellular invasiveness by incorporation of MNPs at the concentration of 50 μg/mL for 48 h (see Figure S12 in SI) is shown. Furthermore, as concerns radiation effects, cell invasiveness is reduced by proton irradiation, whereas photon irradiation seems to have no great effect on cell invasive capacity. Moreover, the value of the invasiveness index in samples treated with MNPs, irradiated with protons and subjected to hyperthermia, seems to be slightly lower than that of only irradiated samples (see Figure 8), as if the hyperthermia treatment also contributed to the decrease in invasive capacity. More details on our data and other literature results are reported in SI.

## 4. Conclusions

In this paper, we propose a combination of treatments for in vitro BxPC3 pancreatic tumor cells: proton irradiation therapy (photons for control), MNPs administration and hyperthermia have been applied in sequence and the effects on clonogenic survival, DSBs, ROS, cell cycle and cell invasion have been investigated. Proton therapy has been delivered using the synchrotron-based clinical facility CNAO in Pavia (Italy) and, for comparison, photon beam irradiation was performed using a 6 MV linear accelerator at INT in Milano (Italy). Hyperthermia was applied by increasing the temperature of the cells’ vial from 37 °C to 42 °C and keeping the final temperature for 0.5 h, using also assisted-Magnetic Fluid Hyperthermia. For the temperature increase by MFH, we used magnetite-based spherical magnetic nanoparticles, coated with dimercaptosuccinic acid that underwent an alternating magnetic field of intensity 15.6 kA/m and frequency 109.8 kHz. The analysis of the experimental results demonstrated that:

(a) The combination of proton therapy, MNPs uptake and hyperthermia is very effective in reducing the clonogenic survival, till levels of a few percent at high (>3 Gy) doses, and gives better results with respect to (photon or proton) irradiation only;
(b) The effect of the combined therapies (PT+MNPs+Hyp) is synergistic, as shown by the change of the fitting model of clonogenic survival data (from linear quadratric to linear), after MNPs are added and Hyp applied;
(c) The number of DNA DSBs is increased at 6 h after the combined PT+MNPs+Hyp treatments, in agreement with clonogenic survival data;
(d) Combined PT, MNPs uptake and Hyp increases the production of ROS; the values of ROS generation after irradiation and following hyperthermia are 0.90 ± 0.09 (photons) and 0.90 ± 0.09 (protons) compared to 0.50 ± 0.05 radiation alone; this also contributes to cytotoxic cellular effects and to a wide variety of lesions including DNA damage;
(e) A radiosensitizing effect of MNPs combined with proton/photon irradiation has been proven by analyzing the dose enhancement factor that, for the combined therapy, resulted in being increased till the values ∼2.8 ± 0.3 (photons) and 2.5 ± 0.3 (protons), estimated at 10% of survival. The DEF values are almost twice the ones for cells subjected to radiations only;
(f) The resulting proton therapy RBE is ∼1.3, as a combination of MNP-induced radiosensitization effects and dose enhancement factor.

Results (d)–(f) have been extended also to the data of an old work [19], where irradiation was made by means of carbon ions. Finally, it is important to note that the present work could be extended to in vivo pre-clinical cases or, in the absence of an experimental facility (as in our specific case), to 3D cellular scaffolds that have been recently demonstrated to properly simulate the in vivo systems [37,38,39]. The proposed novel therapy, once translated to the clinic, could improve the pancreatic cancer treatment by contributing to increasing the survival rate, disease regression and quality of life of patients. Its use for other kinds of tumors and the increasing number of proton therapies installed in hospitals are also envisaged to contribute towards possibly improving the general public healthcare. 

## Figures and Tables

**Table 1:** Summary of the experiments performed in this work and in the previous one [19].

Radiation Type	Clonogenic Survival	DSB	Dose Enhancement	RBE	ROS	Cell Cycle	Cell Invasion
*Protons*	X	X	X	X	X	X	X
*Photons*	X	X	X	/	X	X	X
*Carbon Ions*	[19]	[19]	X	[19]	X	X	X

## Data Availability

The authors confirm that the experimental data supporting the findings of this study are available within the article and its supplementary materials.

## Supplementary Materials

The following supporting information can be downloaded at: https://www.mdpi.com/article/10.3390/nano13050791/s1, Figure S1: Diffraction pattern of MNPs; Figure S2: Curve Temperature vs. Time under the application of an alternating magnetic field; Figure S3: ZFC/FC magnetization curves collected with a magnetic field $\mu_{0}$H = 5 × 10${}^{-3}$ Tesla and hysteresis loop at 260 K. Figure S4: Representative images of 53BP1 and γ-H2AX stained cells; Figure S5: Comparison between the dose enhancement factors of the different radiation beams, measured at 10% and 2% of cell survival; Figure S6: Flow cytometry analysis of cells to detect ROS induction after staining with DCFH-DA; Figure S7: Flow cytometry analysis of BxPC3 cells to detect ROS induction after carbon ion irradiation and following hyperthermia; Figure S8: Flow cytometry analysis of BxPC3 cells to detect ROS induction after proton irradiation and following hyperthermia; Figure S9: Flow cytometry analysis of BxPC3 cells to detect ROS induction after photon irradiation and following hyperthermia; Figure S10: Cell cycle phase distribution of BxPC3 cells with 50 μg/mL-MNPs, exposed to different types of radiation and subsequent hyperthermia; Figure S11: SubG1 cell fraction of BxPC3 cells with 50 μg/mL-MNPs, exposed to different types of radiation and subsequent hyperthermia (both immediately and after longer time periods); Figure S12: Relative invasion index of BxPC3 cells treated with MNPs and exposed to hyperthermia treatment; Figure S13: Relative invasion index of BxPC3 cells exposed to carbon ions, protons and photons, treated or not with MNPs and hyperthermia; Table S1: Cell cycle analysis of BxPC3 cells measured by flow cytometry after 48 h of treatment with MNPs (50 μg/mL).
