# Supplementary material for: Proton Therapy, Magnetic Nanoparticles and Hyperthermia as Combined Treatment for Pancreatic BxPC3 Tumor Cells
Source: Nanomaterials (Basel). 2023 Feb 21;13(5):791. doi: 10.3390/nano13050791 (PMC10005040; doi:10.3390/nano13050791)
Supplement: Supplementary file 1 [file nanomaterials-13-00791-s001.zip › nanomaterials-2208347-supplementary.pdf]

Supplementary Materials: Proton Therapy, Magnetic Nanoparticles and Hyperthermia as Combined Treatment for Pancreatic BxPC3 Tumor Cells

Francesca Brero [1,†], Paola Calzolari [2], Martin Albino [3,4], Antonio Antoccia [5], Paolo Arosio [2], Francesco Berardinelli [5], Daniela Bettega [2], Mario Ciocca [6], Angelica Facoetti [6], Salvatore Gallo [2], Flavia Groppi [2,7], Claudia Innocenti [3,4,†], Anna Laurenzana [8], Cristina Lenardi [2], Silvia Locarno [2], Simone Manenti [2,7], Renato Marchesini [2], Manuel Mariani [9,*], Francesco Orsini [2], Emanuele Pignoli [10], Claudio Sangregorio [3,4,11], Francesca Scavone [8], Ivan Veronese [2] and Alessandro Lascialfari [1,9,*]

1. Details on Materials and Methods

1.1. Detection of reactive oxygen species (ROS)

The BxPC3 cells are incubated with MNPs at 50 μg/ml for 48 h. After this time, aliquots of treated MNP-cells are exposed to hyperthermia treatment (42°C for 30 minutes). The generation of cellular ROS are detected by incubation of cell samples with dichlorodihydrofluorescein diacetate (DCFH-DA), which penetrates the cells and is hydrolysed to DCFH, a nonfluorescent compound that remains trapped within the cells. The different cell samples (MNPs alone, MNPs+Hyperthermia and control) are incubated with 10 μM DCFH-DA in PBS (phosphate-buffered saline) for 45 minutes at 37°C. Following incubation, the samples are put at 4°C overnight. Cells incubated without MNPs are used as negative control and cells treated with 1mM H_2O_2 as a positive control. ROS are assessed by analysing the cells by flow cytometry using the FL1 channel. At least 5×10^4 cells are analysed for each sample. A similar protocol was followed for cell samples treated with MNPs, irradiated, and exposed to hyperthermia treatment.

1.2. Cell cycle analysis

Briefly, immediately after irradiation of cells without MNPs and after irradiation + hyperthermia of cells treated with MNPs: (i) aliquots of cell suspensions are centrifuged at 1800 rpm for 10 minutes; the cell pellets are then resuspended in cold absolute ethyl alcohol and placed at -20°C for subsequent treatments for the flow cytometric analysis of the cell cycle; (ii) aliquots of same cell suspensions are seeded in flasks T25 and placed in the incubator (37°C, 5% CO_2) of the CNAO Radiobiology laboratory. After about 30-34 hours, these samples are transported to the Radiobiology Laboratory of the Physics Department of Milan, where they are trypsinized and the cells are resuspended in culture medium and centrifuged at 1800 rpm for 10 minutes. The cell pellets are then resuspended in cold absolute ethanol and placed at -20°C for subsequent treatments for the flow cytometric analysis of the cell cycle. A similar protocol was followed for cell samples irradiated with photons, immediately and about 24 hours after irradiation and hyperthermia treatment. For cytofluorimetric analysis the cells are treated with Ribonuclease A (100 μg/ml – Sigma Aldrich) for 30 min at 37°C and then stained with propidium iodide (50 μg/ml – Sigma Aldrich) for about 12–14 hours. The flowcytometer BD ACCURI C6 is used for the experiments and at least 4×10^4 cells are counted at each point. The proportion of cells at different phases is gated and calculated using the ModFit Lt software.

1.3. Cell invasion

Cell invasion is measured using the QCM EC Matrix Cell Invasion Assay (Merk Millipore) with 8 µm pore size polycarbonate membrane. About 1.5×10^5 cells are plated in upper wells in serum-free medium. After incubation at 37°C for 24 hours, the non-invading cells are scrubbed off the upper membrane, while invading cells on the lower membrane are stained with crystal violet stain and counted by optical microscopy (x50 magnification). The invasive capacity is measured as the ratio between invading cells and the total number of cells initially seeded, obtained values are normalized to the control ones.

2. Magnetic nanoparticles

2.1. X-ray diffraction

The magnetic cores of the nanoparticles were characterized by X-ray diffraction (fig. S1). All the peaks of the diffraction pattern match the reference pattern of magnetite (red bars), indicating the presence of a single crystalline phase in the sample. The lattice parameter is also compatible with that of magnetite.

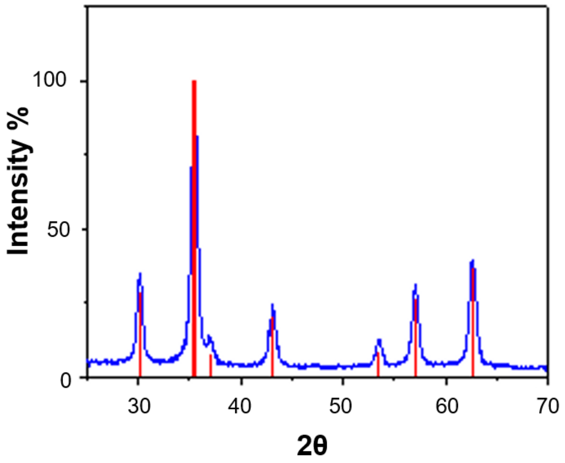

Figure S1. Diffraction pattern of MNPs (blue line) compared with the reference pattern of magnetite (red bars).

2.2. Specific Absorption Rate

The hyperthermic efficiency was estimated by evaluating the specific absorption rate (SAR), by recording (see fig. S2) the temperature rise of a MNPs solution sample (0.98 mg/mL) under the effect of an alternating magnetic field (f = 183 kHz, H= 17 kA/m). The value obtained by the initial slope method is SAR = 225 W/g_{Fe}.

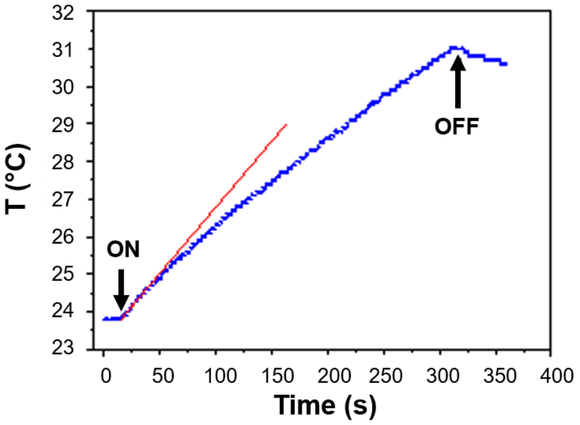

Figure S2. Temperature increases upon time under the application of an alternating magnetic field. The red line denotes the initial slope of the curve, used for the SAR estimate. The arrows indicate the on/off switching times of the field.

2.3. Magnetic measurements

The field and temperature dependencies of the MNPs magnetization was evaluated by recording the ZFC-FC curves with μ_0H = 5×10^{-3} T and the hysteresis loop at 260 K (see fig. S3). The maximum of the ZFC-FC curve is broadened around the end of the measuring temperature range, suggesting that MNPs are in a sort of transition between

"blocked/unblocked" (superparamagnetic) regimes at 250 K. The absence of hysteretic behavior at 260 K indicated that the MNPs are in the superparamagnetic regime at room temperatures.

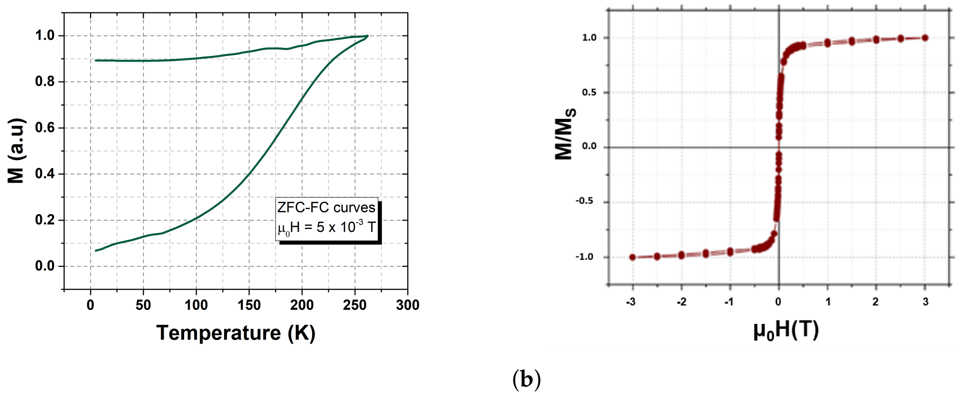

Figure S3. (a) ZFC/FC magnetization curves collected with a magnetic field $\mu_0 H$ = 5×10^{-3} Tesla and (b) hysteresis loop at 260 K.

3. Biological Effects

3.1. MNPs and Hyperthermia Cell Toxicity

The plating efficiency of BxPC3 cells decreases from about 50% to 23% when MNPs are added, due to the MNPs toxicity at 14 days. A further decrease of PE to about 6% is observed when hyperthermia is also applied. Therefore, hyperthermia in cells treated with MNPs shows a further effect of cell mortality compared to treatment with MNPs alone. Similar results were reported in the work of Russel et al. [1], that investigated the toxic effect of SPIONs using the clonogenic assay. The obtained values of plating efficiencies after 24 hours of exposure to SPIONs (Superparamagnetic iron oxide nanoparticles), having a concentration equal to 23.5 $\mu g/ml$, showed a significant decrease in plating efficiencies of U87 and HEPG2 cells. The work of Khoei et al. [2] showed that the presence of IUdR/MNPs (SPIONs, as a carrier of 5-iodo-2-deoxyuridine, in short IUdR) in monolayer culture of U87MG cells increased the toxicity; moreover, the reduction of the plating efficiencies due to the combination effect of IUdR/MNPs and MFH was significantly more than that of each treatment alone.

3.2. Double strand breaks studies

The kinetics of DNA double strand breaks (DSBs) rejoining has been evaluated by means of γ-H2AX and 53BP1 foci formation by immunofluorescence analysis (see fig. S4).

3.3. Dose enhancement factor (DEF)

The dose enhancement factor, i.e. the ratio between the radiation doses used alone and in conjunction with the MNPs in order to obtain the same biological endpoint, has been evaluated for protons, photons and carbon ions (fig. S5).

3.4. Detection of reactive oxygen species

Reactive oxygen species (ROS) generation has been evaluated for different cell samples, incubating with 50 μg/ml-MNPs and treated with hyperthermia (fig. S6), irradiated with carbon ions (fig. S7), protons (fig. S8) and photons (fig. S9). In literature, several studies have suggested that iron oxide nanoparticles induce ROS formation [3–7]. It has also been described that iron oxide MNPs may generate ROS, which could be enhanced by local hyperthermic effects when MNPs are exposed to a alternating magnetic field [8–11].

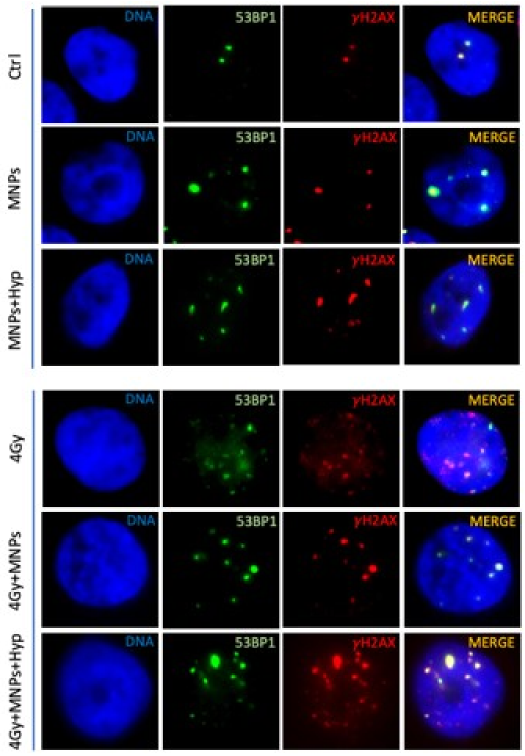

Figure S4. Representative images of 53BP1 (green) and γ-H2AX (red) stained cells. Unirradiated cells treated with MNPs and MNPs+Hyp (upper figure) and Proton irradiated cells in combination with MNPs and MNPs+Hyp (4Gy; fixed 6h after irradiation - lower figure)

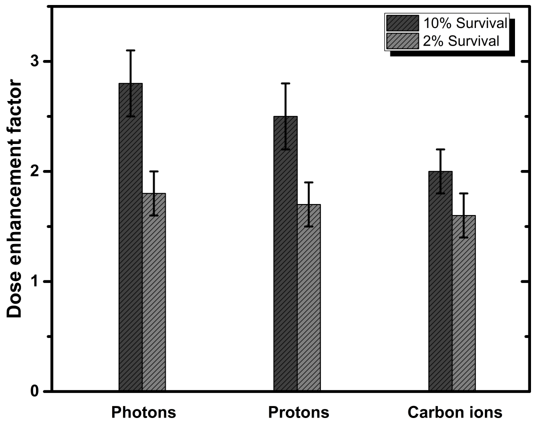

Figure S5. Comparison between the dose enhancement factors of the different radiation beams, measured at 10% and 2% of cell survival. Error bars represent one standard error of the mean of 4 independent experiments.

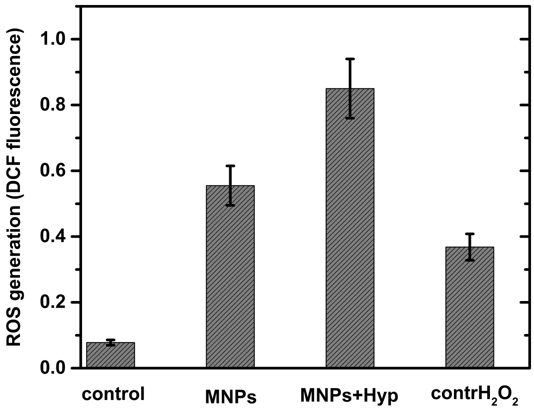

Figure S6. Flow cytometry analysis of cells to detect ROS induction after staining with DCFH-DA. Mean ± SD (3 experiments). Control is negative control, untreated cells; contrH_2O_2 is positive control, cells incubated with 1mM H_2O_2; MNPs, cells with MNPs-50 μg/ml; MNPs+Hyp: cells with MNPs-50 μg/ml and hyperthermia treatment.

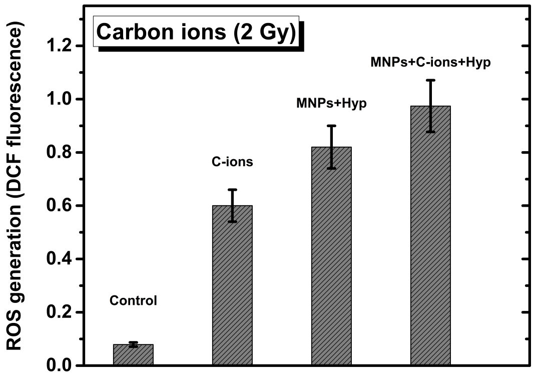

Figure S7. Flow cytometry analysis of BxPC3 cells to detect ROS induction after carbon ion irradiation and following hyperthermia. Control: untreated cells; C-ions: cells irradiated with 2 Gy of carbon ions; MNPs+Hyp: cells with MNP-50 μg/ml and hyperthermia treatment; MNPs+C-ions+Hyp: cells with MNPs-50 μg/ml, irradiated with 2 Gy carbon ions and hyperthermia treatment. Mean ± SD (2 experiments).

3.5. Cell cycle analysis

The data reported in Table S1 show that there are no significant variations in the values of the cell phases in samples treated with MNPs for 48 h (50 μg/ml), without irradiation and hyperthermia treatments, and untreated (control). This result indicates that MNPs incorporated into BxPC3 cells does not alter the cell cycle. Similar results have been found in several works with different cell lines. Calero et al. with breast cancer cells, MCF-7, incubated with 0.4 mg/ml-SPION for 24 h [3], Ma et al. with human lung cancer cells, NCI-H460, incubated with 400 μg/ml of magnetic nanoparticles clusters, MNCs, for 24 hours [12] and Xia et al. with myelodysplastic syndrome cells, SKM-1, incubated for 24 hours with MNPs [13].

Sample	G1 phase (%)	S phase (%)	G2+M phase (%)	subG1 phase (%)
control	60,3 ± 3,7	25,2 ± 1,8	14,5 ± 1.3	-
MNPs-50 µg/ml	58,7 ± 4,5	22,8 ± 2,3	13,2 ± 1,3	5,3 ± 0,5

Table S1. Cell cycle analysis of BxPC3 cells measured by flow cytometry after 48 h of treatment with MNPs (50μg/ml). The results for each sample are averaged over three independent experiments.

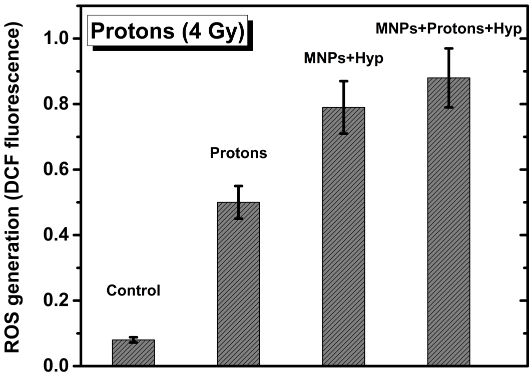

Figure S8. Flow cytometry analysis of BxPC3 cells to detect ROS induction after proton irradiation and following hyperthermia. Control: untreated cells; Protons: cells irradiated with 4 Gy of protons; MNPs+Hyp: cells with MNP-50 μg/ml and hyperthermia treatment; MNPs+Protons+Hyp: cells with MNP-50 μg/ml, irradiated with 4 Gy of protons and hyperthermia treatment. Mean $\pm$ SD (2 experiments).

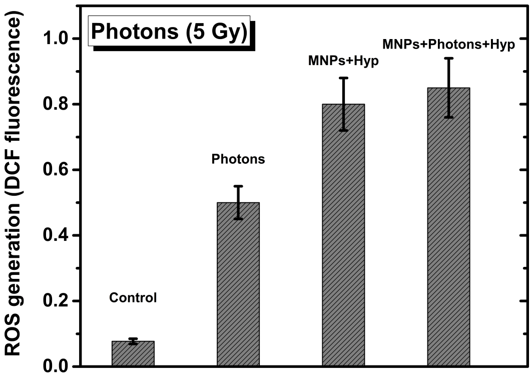

Figure S9. Flow cytometry analysis of BxPC3 cells to detect ROS induction after photon irradiation and following hyperthermia. Control: untreated cells; Photons: cells irradiated with 5 Gy of photons; MNPs+Hyp: cells with MNP-50 μg/ml and hyperthermia treatment; MNPs+Photons+Hyp: cells with MNP-50 μg/ml, irradiated with 5 Gy of photons and hyperthermia treatment. Mean $\pm$ SD (2 experiments).

3.5.1. Radiation alone

Exposure of BxPC3 cells to a dose of 2 Gy of carbon ions, 4 Gy of protons and 5 Gy of photons resulted in an increase of cells in the G2/M phase. In fact, these samples, treated with radiation alone, show a significant percentage of cells in the G2/M phase even after a time of about 34 hours from irradiation (see Fig. S10). This effect induced by the radiation is known in literature and is called "G2/M cell cycle arrest"; the intensity and duration of this cell block in the G2/M phase depend on the cell line, the type of radiation, the radiation dose and the time elapsed since irradiation. Naumann et al. [14] with the same cell line (BxPC3), in cells irradiated with a dose of 4 Gy of photons found an increased percentage of cells "blocked" in the G2/M phase after a period of 24 hours from exposure to radiation. Our results showed that irradiation with carbons, protons and photons induced G2/M arrest indicating a high level of DNA damage. Cells with unrepaired or poorly repaired DNA damage can persist in the G2/M phase leading to genomic instability, cell death and therefore to an inhibition of cell proliferation.

3.5.2. MNPs and hyperthermia treatments

The results of the cell cycle analysis after MNPs and hyperthermia treatments, without irradiation, show an increase of the S phase, about 36% compared to a value of about 25% for the samples without MNPs and hyperthermia treatments (see fig. S10). It is unknown how hyperthermia affects the cell cycle distribution, but these data seem to confirm that S-phase cell accumulation following hyperthermia treatment results in higher cell mortality. Ma et al. [12] found that lung cancer cells (NCI-H460) treated with magnetic nanoparticle clusters, irradiated with a dose of 4 Gy of 6 MV X-ray and subjected to magnetic hyperthermia after a 24-hour period show a significant increase in the S phase compared to control samples with or without MNPs and which did not undergo hyperthermia treatment. For the MNPs+radiation+hyperthermia samples, the percentages of the S and G2 / M phases are practically the same, around 21%; perhaps any non-increase in phase S could be "counterbalanced" by the increase in cells in the G2 / M phase due to radiation (see Figure S10).

3.5.3. Sub-G1 cell fraction

The samples treated with MNPs show a small percentage (about 5%) of apoptotic cells; the subG1 population, cell fragments, is a marker of apoptosis [6,15] due to the cytotoxicity of the nanoparticles (see Table S1 and Figures S7,S8,S9). The cell samples treated with MNPs+hyperthermia and MNPs+radiation+hyperthermia (see Figure S11) show a percentage of apoptotic cells around the value of 8–12% (immediately after irradiation and after 24-34 hour post irradiation); this increase appears to be due the combination of the treatments.

3.6. Cell invasion

Figure S12 shows the relative invasion index of BxPC3 cells untreated or treated with MNP-50 μg/ml for 48 hours and subsequent hyperthermia treatment; the heating is applied for 30 minutes at 42°C.

Cell invasion is related to cell migration, and defines the ability of cells to become motile and to navigate through the extracellular matrix within a tissue or to infiltrate neighbouring tissues. Cancer cells that become invasive may disseminate to secondary sites and form metastases. Tumor cell invasion is an essential step of cancer progression that is associated with an enhanced capability of tumor cells to degrade extracellular matrix components. The application of nanomedicine using nanoparticles - MNPs - could be an alternative approach to the treatment of cancers through the inhibition of cell invasion.
The values of the samples untreated or treated with the nanoparticles are similar to one other, thus showing no effect on cellular invasiveness by incorporation of MPNs at this concentration. Cells treated with MNPs and subjected to hyperthermia show a slight decrease in the cellular invasiveness index. As concerns radiation effects, the relative

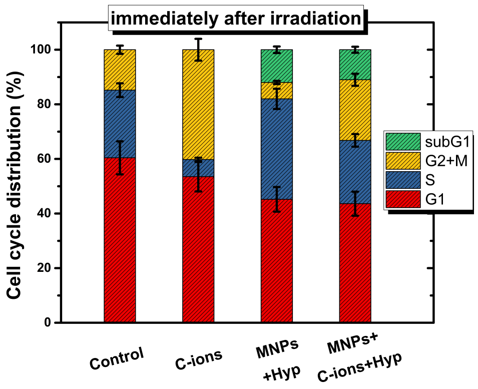

(**a**)

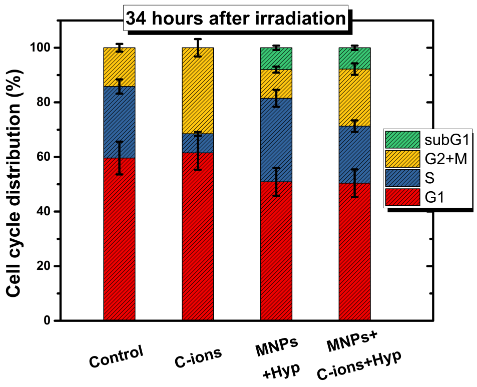

(**b**)

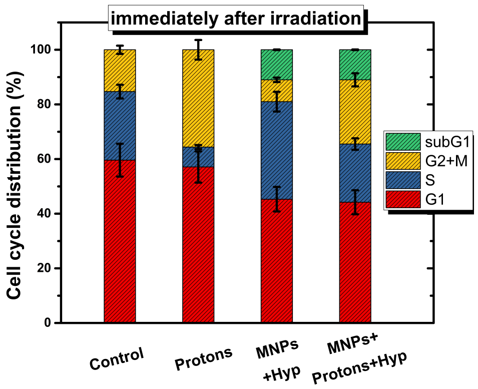

(**c**)

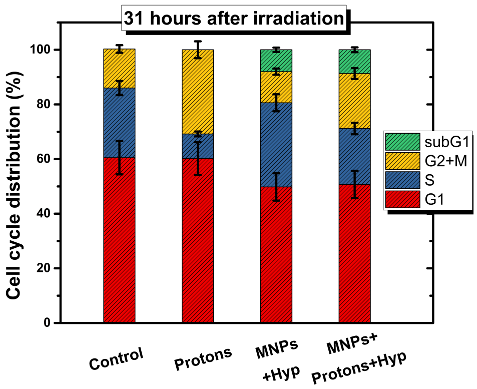

(**d**)

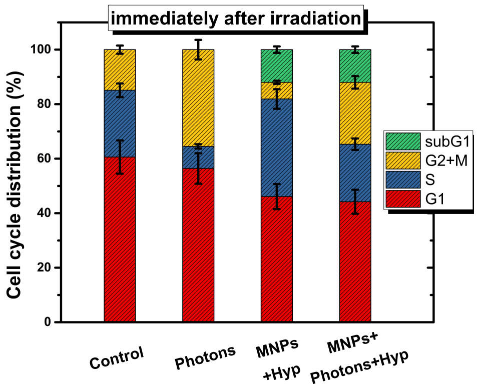

(**e**)

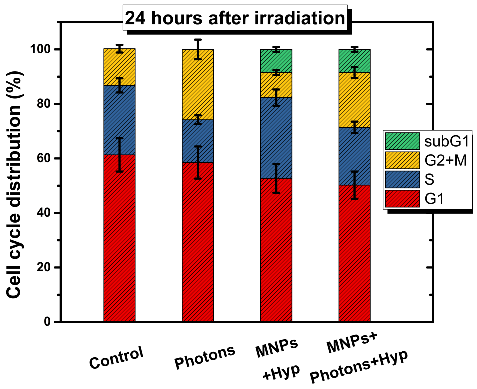

(**f**)

Figure S10. Cell cycle phase distribution of BxPC3 cells with 50 μg/ml-MNPs, exposed to different types of radiation and subsequent hyperthermia. Control: untreated cells; C-ions/Protons/Photons: cells irradiated with 2 Gy of carbon ions or 4 Gy of protons or 5 Gy of photons; MNPs+Hyp: cells with MNP-50 μg/ml and hyperthermia treatment; MNPs+C-ions/Protons/Photons+Hyp: cells with MNP-50 μg/ml, irradiation and hyperthermia treatment. (Sub G1 cell fraction as marker for cell fraction in apoptosis).

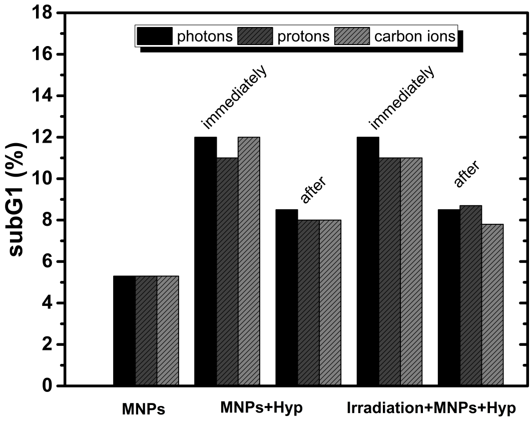

Figure S11. SubG1 cell fraction of BxPC3 cells with 50 μg/ml-MNPs, exposed to different types of radiation and subsequent hyperthermia (both immediately and after longer time periods). MNPs: cells with MNP-50 μg/ml; MNPs+Hyp: cells with MNP-50 μg/ml and hyperthermia treatment; Irradiation+MNPs+Hyp: cells with MNP-50 μg/ml, irradiation and hyperthermia treatment.

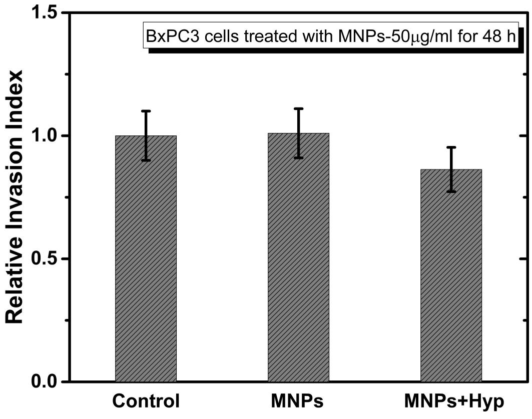

Figure S12. The relative invasion index of BxPC3 cells treated with MNPs and exposed to hyperthermia treatment. Control: untreated cells; MNPs: cells with MNP-50 microgrammi/ml; MNPs+Hyp: cells with MNP-50 μg/ml and hyperthermia treatment. Error bars represent one standard error of the mean of 3 experiments.

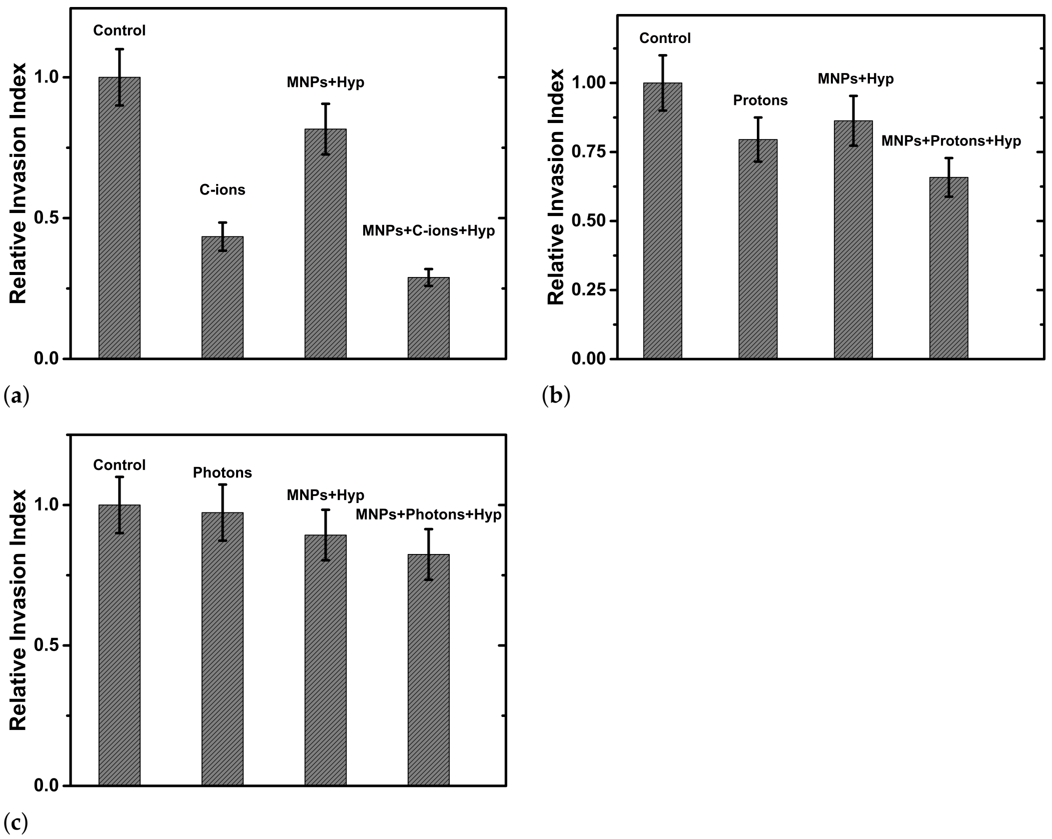

Figure S13. The relative invasion index of BxPC3 cells exposed to carbon ions (a), protons (b) and photons (c) treated or not with MNPs and hyperthermia. Control: untreated cells; C-ions/Protons/Photons: cells irradiated with 2 Gy of carbon ions or 4 Gy of protons or 5 Gy of photons; MNPs+Hyp: cells with MNPs-50 μg/ml and hyperthermia treatment; MNPs+C-ions/Protons/Photons+Hyp: cells with MNP-50 μg/ml, irradiation and hyperthermia treatment. Error bars represent one standard error of the mean of 2 experiments.

invasion index of BxPC3 cells exposed to photons (at dose of 5 Gy - fig. S13c), protons (at dose of 4 Gy - fig. S13b) and carbon ions (at dose of 2Gy - fig. S13a) treated or not with MNPs-50 $\mu g/ml$ for 48 hours and subsequent hyperthermia treatment is shown. Reported data are the mean of two independent experiments.

A reduction in the invasion rate is also observed in cells irradiated with carbon ions treated with MNPs or untreated. Cell invasiveness is less reduced by proton irradiation whereas photon irradiation seems to have no great effect on cell invasive capacity. Therefore our data seem to indicate that carbon beam irradiation and, to a lesser effect, the irradiation with protons suppressed invasiveness potential more effectively than photon irradiation. The value of the invasiveness index in samples treated with MNPs, irradiated with protons and carbon ions and subjected to hyperthermia seems to be slightly lower than that of only irradiated samples, as if the hyperthermia treatment also contributed to the decrease in invasive capacity. Several studies demonstrated that ionizing radiation might promote migration and invasion of tumor cells by various pathways and other studies reported that carbon ions and photon radiation produced different effects on the migration and invasiveness of tumor cells [16,17]. Fujita et al. [18,19] reported that carbon ions suppress migration and invasion in human pancreatic carcinoma cells (the BxPC3 cell line used in our experiments) via the inhibition of the activity of Rac1 through Ub-mediated proteosomal degradation. There are only few reports studying the effects of magnetic-nanoparticle altered cell invasion. Veiseh et al. [20,21] explore a new approach of nanoparticle-based therapy for treatment of cancer through the inhibition of tumor cell invasion, a strategy

particularly suitable for highly invasive cancer as glioma. C6 rat glioma cells treated with nanoparticles with polymer coating (PEG) and chlorotoxin (CTX)-PEG-CTX show inhibition of cell invasion at the highest tested exposure concentration, while cells with nanoparticles with PEG but not CTX show no evident cell invasion inhibition. Li et al. [22] show that the invasiveness of lung cancer cells, A549, is severely impaired by the combined treatment of cold atmospheric plasma (CAP) and nanoparticles (iron-oxide-based, MNPs), while cells with only MNPs show a lower cell invasion inhibition. The objectives reported in the work of Sasaki et al. [23] are different from those of our study. Indeed the aim of their study is to evaluate cellular invasion ability of normal human fibroblasts labeled with MNPs coated with chitosan and using magnetic forces; this may accelerate the development of cellular strategies for the repair or replacement of normal tissue.

1. Russell, E.; Dunne, V.; Russell, B.; Mohamud, H.; Ghita, M.; McMahon, S.J.; Butterworth, K.T.; Schettino, G.; McGarry, C.K.; Prise, K.M. Impact of superparamagnetic iron oxide nanoparticles on in vitro and in vivo radiosensitisation of cancer cells. *Radiation Oncology* **2021**, *16*, 1–16.
2. Khoei, S.; Hosseini, V.; Hosseini, M.; Khoee, S.; Shirvalilou, S.; Mahdavi, S.R.; Pirayesh Islamian, J. Enhancement of radio-thermo-sensitivity of 5-iodo-2-deoxyuridine-loaded polymeric-coated magnetic nanoparticles triggers apoptosis in U87MG human glioblastoma cancer cell line. *Cellular and Molecular Bioengineering* **2021**, *14*, 365–377.
3. Calero, M.; Chiappi, M.; Lazaro-Carrillo, A.; Rodríguez, M.J.; Chichón, F.J.; Crosbie-Staunton, K.; Prina-Mello, A.; Volkov, Y.; Villanueva, A.; Carrascosa, J.L. Characterization of interaction of magnetic nanoparticles with breast cancer cells. *Journal of nanobiotechnology* **2015**, *13*, 1–15.
4. Singh, N.; Jenkins, G.J.; Asadi, R.; Doak, S.H. Potential toxicity of superparamagnetic iron oxide nanoparticles (SPION). *Nano reviews* **2010**, *1*, 5358.
5. Watanabe, M.; Yoneda, M.; Morohashi, A.; Hori, Y.; Okamoto, D.; Sato, A.; Kurioka, D.; Nittami, T.; Hirokawa, Y.; Shiraishi, T.; et al. Effects of Fe3O4 magnetic nanoparticles on A549 cells. *International journal of molecular sciences* **2013**, *14*, 15546–15560.
6. Feng, Q.; Liu, Y.; Huang, J.; Chen, K.; Huang, J.; Xiao, K. Uptake, distribution, clearance, and toxicity of iron oxide nanoparticles with different sizes and coatings. *Scientific reports* **2018**, *8*, 1–13.
7. Naqvi, S.; Samim, M.; Abdin, M.; Ahmed, F.J.; Maitra, A.; Prashant, C.; Dinda, A.K. Concentration-dependent toxicity of iron oxide nanoparticles mediated by increased oxidative stress. *International journal of nanomedicine* **2010**, *5*, 983.
8. Sanhaji, M.; Göring, J.; Couleaud, P.; Aires, A.; Cortajarena, A.L.; Courty, J.; Prina-Mello, A.; Stapf, M.; Ludwig, R.; Volkov, Y.; et al. The phenotype of target pancreatic cancer cells influences cell death by magnetic hyperthermia with nanoparticles carrying gemicitabine and the pseudo-peptide NucAnt. *Nanomedicine: Nanotechnology, Biology and Medicine* **2019**, *20*, 101983. https://doi.org/https://doi.org/10.1016/j.nano.2018.12.019.
9. Ludwig, R.; Teran, F.J.; Teichgraeber, U.; Hilger, I. Nanoparticle-based hyperthermia distinctly impacts production of ROS, expression of Ki-67, TOP2A, and TPX2, and induction of apoptosis in pancreatic cancer. *International journal of nanomedicine* **2017**, *12*, 1009.
10. Wydra, R.J.; Rychahou, P.G.; Evers, B.M.; Anderson, K.W.; Dziubla, T.D.; Hilt, J.Z. The role of ROS generation from magnetic nanoparticles in an alternating magnetic field on cytotoxicity. *Acta biomaterialia* **2015**, *25*, 284–290.
11. Mejías, R.; Hernandez Flores, P.; Talelli, M.; Tajada-Herráiz, J.L.; Brollo, M.E.; Portilla, Y.; Morales, M.P.; Barber, D.F. Cell-promoted nanoparticle aggregation decreases nanoparticle-induced hyperthermia under an alternating magnetic field independently of nanoparticle coating, core size, and subcellular localization. *ACS applied materials & interfaces* **2018**, *11*, 340–355.
12. Ma, J.; Zhang, Z.; Zhang, Z.; Huang, J.; Qin, Y.; Li, X.; Liu, H.; Yang, K.; Wu, G. Magnetic nanoparticle clusters radiosensitise human nasopharyngeal and lung cancer cells after alternating magnetic field treatment. *International Journal of Hyperthermia* **2015**, *31*, 800–812.
13. Xia, G.; Chen, B.; Ding, J.; Gao, C.; Lu, H.; Shao, Z.; Gao, F.; Wang, X. Effect of magnetic Fe3O4 nanoparticles with 2-methoxyestradiol on the cell-cycle progression and apoptosis of myelodysplastic syndrome cells. *International Journal of Nanomedicine* **2011**, *6*, 1921.
14. Naumann, P.; Liermann, J.; Fortunato, F.; Schmid, T.E.; Weber, K.J.; Debus, J.; Combs, S.E. Sulforaphane enhances irradiation effects in terms of perturbed cell cycle progression and increased DNA damage in pancreatic cancer cells. *PLoS One* **2017**, *12*, e0180940.

15. Sanhaji, M.; Göring, J.; Couleaud, P.; Aires, A.; Cortajarena, A.L.; Courty, J.; Prina-Mello, A.; Stapf, M.; Ludwig, R.; Volkov, Y.; et al. The phenotype of target pancreatic cancer cells influences cell death by magnetic hyperthermia with nanoparticles carrying gemicitabine and the pseudo-peptide NucAnt. *Nanomedicine: Nanotechnology, Biology and Medicine* **2019**, *20*, 101983.
16. Moncharmont, C.; Levy, A.; Guy, J.B.; Falk, A.T.; Guilbert, M.; Trone, J.C.; Alphonse, G.; Gilormini, M.; Ardail, D.; Toillon, R.A.; et al. Radiation-enhanced cell migration/invasion process: a review. *Critical reviews in oncology/hematology* **2014**, *92*, 133–142.
17. Fujita, M.; Yamada, S.; Imai, T. Irradiation induces diverse changes in invasive potential in cancer cell lines. In Proceedings of the Seminars in Cancer Biology. Elsevier, 2015, Vol. 35, pp. 45–52.
18. Fujita, M.; Otsuka, Y.; Imadome, K.; Endo, S.; Yamada, S.; Imai, T. Carbon-ion radiation enhances migration ability and invasiveness of the pancreatic cancer cell, PANC-1, in vitro. *Cancer Science* **2012**, *103*, 677–683.
19. Fujita, M.; Imadome, K.; Shoji, Y.; Isozaki, T.; Endo, S.; Yamada, S.; Imai, T. Carbon-ion irradiation suppresses migration and invasiveness of human pancreatic carcinoma cells MIAPaCa-2 via Rac1 and RhoA degradation. *International Journal of Radiation Oncology* Biology* Physics* **2015**, *93*, 173–180.
20. Veiseh, O.; Gunn, J.W.; Kievit, F.M.; Sun, C.; Fang, C.; Lee, J.S.; Zhang, M. Inhibition of tumor-cell invasion with chlorotoxin-bound superparamagnetic nanoparticles. *Small* **2009**, *5*, 256–264.
21. Veiseh, O.; Kievit, F.M.; Ellenbogen, R.G.; Zhang, M. Cancer cell invasion: treatment and monitoring opportunities in nanomedicine. *Advanced drug delivery reviews* **2011**, *63*, 582–596.
22. Li, W.; Yu, H.; Ding, D.; Chen, Z.; Wang, Y.; Wang, S.; Li, X.; Keidar, M.; Zhang, W. Cold atmospheric plasma and iron oxide-based magnetic nanoparticles for synergetic lung cancer therapy. *Free Radical Biology and Medicine* **2019**, *130*, 71–81.
23. Sasaki, T.; Iwasaki, N.; Kohno, K.; Kishimoto, M.; Majima, T.; Nishimura, S.I.; Minami, A. Magnetic nanoparticles for improving cell invasion in tissue engineering. *Journal of Biomedical Materials Research Part A: An Official Journal of The Society for Biomaterials, The Japanese Society for Biomaterials, and The Australian Society for Biomaterials and the Korean Society for Biomaterials* **2008**, *86*, 969–978.
